# Attenuation of Oxidative Stress of Erythrocytes by Plant-Derived Flavonoids, Orientin and Luteolin

**DOI:** 10.1155/2016/3401269

**Published:** 2016-02-04

**Authors:** Fang An, Shulin Wang, Danhua Yuan, Yuewen Gong, Shuhua Wang

**Affiliations:** ^1^Graduate Faculty, Hebei North University, Zhangjiakou, Hebei 075000, China; ^2^Department of Pharmacy, Hebei North University, Zhangjiakou, Hebei 075000, China; ^3^Faculty of Pharmacy, University of Manitoba, 750 McDermot Avenue, Winnipeg, MB, Canada R3E 0T5

## Abstract

Erythrocytes are easy to be injured by oxidative stress in their lifespan. Although there are several chemicals such as vitamin C (VC) that would be able to reduce oxidative stress, natural herbal products still remain an interesting research area. The current study investigated the effects of two plant-derived flavonoids, orientin and luteolin, on erythrocytes and their possible mechanisms. This experiment was divided into nine groups, which were normal group, model group, VC control group, and treated groups with different doses of orientin and luteolin (10, 20, and 40 *μ*g/mL), respectively. Hemolysis rate was determined by spectrophotometry. Antioxidative enzyme and products were evaluated by different methods. Erythrocyte cell surface and cellular structure were observed with scanning or transmission electron microscope, respectively. Oxidative stress induced significant increase in hemolysis rate of erythrocytes. Orientin or luteolin ameliorated hemolysis of erythrocytes in oxidative stress in a dose-dependent manner. Both orientin and luteolin reduced oxidative products and increased antioxidative enzyme activities. Moreover, orientin and luteolin attenuated oxidative stress induced damage of erythrocyte cell surface morphology and cellular structure. In conclusion, orientin and luteolin could protect human erythrocytes from oxidative damage by attenuating oxidative stress, protecting antioxidative enzyme activities, and preserving integrity of erythrocyte structure.

## 1. Introduction

Erythrocytes are the most abundant visible components in the blood system. They usually have a lifespan of 120 days [1]. Reduced number of erythrocytes can cause anemia and lack of oxygen [2]. Since erythrocytes do not contain the nuclei and mitochondria, oxidative stress usually causes membrane lipid peroxidation. Moreover, erythrocyte membranes are rich in unsaturated fats and hemoglobin contains more iron molecules [3]. Both of these are powerful catalysts of free radical reactions. So erythrocytes are sensitive to oxidative damage from reactive oxygen species (ROS) [4]. Therefore, eliminating excess free radicals could reduce membrane lipid peroxidation and protect erythrocytes from oxidative injury.

At present, there are few known drugs that can prevent erythrocytes from oxidative injury. But chemical components extracted from plants have shown some promising trend especially herbs used in China [5]. Flavonoid is one of the chemical components extracted from stem, leaf, and flower of a wide variety of plants, which has a broad spectrum of pharmacological activities and low toxicity [6, 7]. For example, anthocyanins isolated from* the red bayberry* have been shown to decrease the production of ROS and prevent oxidative damage to islet cells [8].

**Figure 1:**
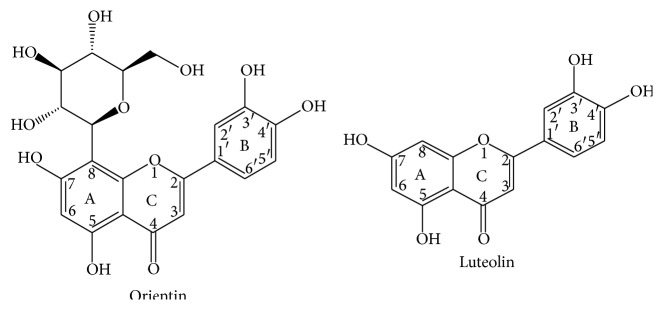
The structure of orientin and luteolin.

From our previous studies, we have identified two flavonoids from our local herbal plant (*Trollius chinensis Bunge*). Orientin belongs to the family of flavonoid glycosides and luteolin belongs to the flavonoid aglycone class [9]. Both of them have been demonstrated to have antioxidative activity in ischemic myocardial disease [10–12]. Their roles in prevention of erythrocytes from oxidative stress are still not completely understood. The current study is to investigate whether orientin and luteolin can protect erythrocytes from oxidative injury and whether there are some differences between these two compounds with different chemical structure (Figure 1).

## 2. Materials and Methods

### 2.1. Preparation of Erythrocyte Suspension

Blood samples from healthy donors (Zhangjiakou Center Blood Stations) were centrifuged at 4°C, 2500 rpm for 10 min, to separate erythrocytes from the blood cells. The erythrocytes were washed three times with phosphate buffer saline (PBS) (pH 7.4) and resuspended at 2% in PBS.

### 2.2. Erythrocytes Oxidative Damage Model

2% erythrocyte suspension (2.0 mL) was added to H_2_O_2_ (2.0 mL) at different concentrations and the final concentrations of H_2_O_2_ were 100, 200, 300, 400, and 500 mM, respectively. The group without H_2_O_2_ was the control group. Erythrocytes were taken from each tube every 30 min after the incubation at 37°C, diluted 10-fold with 0.9% saline or distilled water (ddH_2_O), and then centrifuged at 2500 rpm for 10 min at 4°C. The supernatants were collected to measure the absorbance value (UV-9100 UV-Vis Spectrophotometer, Beijing Lab Tech Instrument Co., Ltd., Beijing, China) at 412 nm. The final hemolysis rate was determined by the final concentration of H_2_O_2_ and incubation time with the following formula: hemolysis rate (%) = *A*/*A*_0_ × 100% (*A*, absorbance value of sample diluted with saline; *A*_0_, absorbance value of sample diluted with ddH_2_O) [13, 14].

### 2.3. Treatment of Erythrocytes with Different Drugs

Orientin (purity ≥ 98.6%) and luteolin (purity ≥ 98.6%) were purchased from Tianjin Party Ltd. and the purity was determined by high performance liquid chromatography (HPLC) and structure was confirmed by 1hydrogen-nuclear magnetic resonance (^1^H-NMR) [9]. Both orientin and luteolin were dissolved in dimethyl sulfoxide (DMSO) and then diluted with CH_3_OH. The concentrations of DMSO and CH_3_OH were maintained at 0.01% and 0.8% of final concentrations, respectively. This experiment was divided into nine groups, which were normal group, model group, VC control group, and treated groups with different doses of orientin and luteolin (10, 20, and 40 *μ*g/mL), respectively. For normal group and model group, erythrocytes were incubated with 100 *μ*L solution of DMSO and CH_3_OH, and erythrocytes were incubated with same dose of different drugs in other groups. Pretreatment of erythrocytes of each group was incubated at 37°C for 30 minutes. At the end of incubation, H_2_O_2_ was added to the solution in model or each treatment group and 0.9% saline was added to the normal group. Erythrocytes were then incubated at 37°C for 1.5 hours and the final hemolysis rate was calculated as indicated above.

### 2.4. Determination of Oxidative Stress in Erythrocytes

Oxidative stress in erythrocytes was determined by measuring enzymatic levels of superoxide dismutase (SOD), catalase (CAT), glutathione peroxidase (GSH-Px), and adenosine trip phosphate system (ATPase). Moreover, ROS and malondialdehyde (MDA) were also measured. After establishment of erythrocyte oxidative damage model, the concentrations of the protein were measured by Bradford protein assay kit from Beyotime Institute of Biotechnology (Shanghai, China). Cytoplasmic proteins were employed for determination of activities of SOD, CAT, GSH-Px, and ATPase. MDA in erythrocyte cytoplasm was extracted using the methods of kits. All these parameters were measured according to the instructions of the available kits from Nanjing Jiancheng Bioengineering Institute (Nanjing, China, with batch numbers: 20120417 for total SOD, 20120419 for CAT, 20120417 for GSH-Px, 20120420 for ATP, and 20120417 for MDA, resp.).

In the paper, the nonpolar 2,7-dichlorofluorescin diacetate (DCFH-DA) was used as a probe to investigate the level of ROS. DCFH-DA has no fluorescence itself; however, it could pass through the cell membrane freely and then was hydrolyzed by cellular esterases into DCFH. The intracellular DCFH could be easily oxidized by ROS into fluorescent dichlorofluorescein (DCH). Thus the ROS generation would be measured by the determination of DCF fluorescence intensity. At the end of treatment, 2% erythrocyte suspension was incubated with 10 mM DCFH-DA at 37°C for 30 min and then washed twice with PBS. Finally, according to the kit from Beyotime Institute of Biotechnology (Shanghai, China), the ROS level was determined in a microplate reader with an excitation wavelength of 485 nm and an emission wavelength of 535 nm.

### 2.5. Preparation and Examination of Erythrocytes for Electron Microscope

Erythrocytes were first centrifuged at 2500 rpm for 10 min at 4°C. The pelleted cells were then mixed with precooled phosphate buffer solution (PBS, pH 7.4, 5 mM Na_2_HPO_4_, 0.1 mM PMSF, and pH 8.0) at ratio 1 : 30 (v/v) and incubated at 4°C for 1 h. The mixture was centrifuged at 4°C, 15000 rpm for 20 min. The pellet was washed three times with cold PBS and centrifugated at the end of each wash at 4°C, 19000 rpm for 20 min. The final pellet was resuspended in cold PBS and the protein concentration was measured by BCA protein assay kit from Beyotime Institute of Biotechnology (Shanghai, China). Erythrocyte membrane protein solution was preserved at concentration of 1 mg/mL in −80°C [15, 16].

For examination of erythrocyte membrane surface morphology, erythrocytes from oxidative stress samples were mixed with 2% glutaraldehyde at ratio 1 : 100 (v/v) and then fixed overnight. After being washed 3 times with cold PBS, the samples were then placed in an ion sputtering device (Hitachi, Japan) to be sprayed with metal after drying and then photographed using the S-3400N scanning electron microscope (SEM, Hitachi) [17].

For examination of erythrocyte membrane skeleton structure, erythrocytes from oxidative stress samples were mixed with NaPi solution (5 mM, pH 7.0) containing 2.5% (W/V) TritonX-100 at 1 : 4 ratio (v/v) and then incubated at 0°C for 1 h. The mixture was dropped onto a copper grid (200 mesh), washed three times with ddH_2_O, and exposed to 1% uranyl acetate for 5–10 s for negative staining. The H-7500 transmission electron microscope (TEM, Hitachi, Japan) was used to observe the erythrocyte membrane skeletal structure after drying with filter paper.

### 2.6. Statistical Analysis

Statistical evaluations were carried out using Statistical Package for Social Sciences (SPSS for Windows, version 17.0). All values were expressed as the mean ± standard deviation (SD). Differences between groups were analyzed by Student's* t*-test. For all tests, *p* values of less than 0.05 were considered significant.

## 3. Investigations and Results

### 3.1. Erythrocyte Oxidative Stress Models

**Figure 2:**
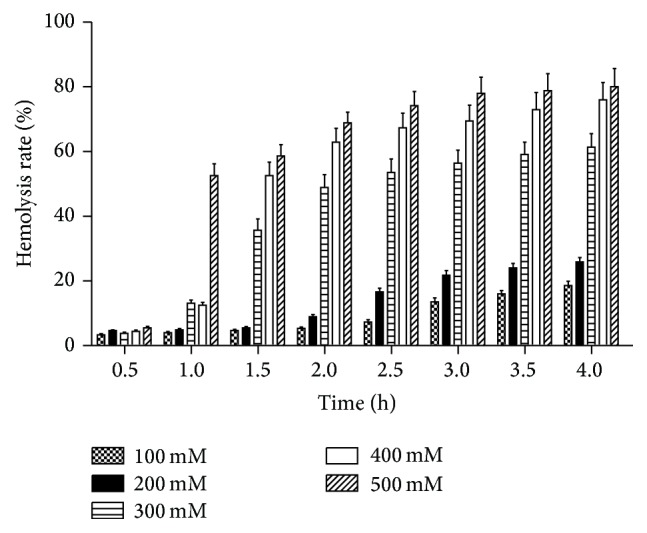
Hemolysis of human erythrocytes. It shows hemolysis rate of human erythrocytes induced by different concentrations of H_2_O_2_ at different time intervals. Data are presented as mean ± SD from six independent experiments.

Erythrocyte oxidative stress was measured by erythrocyte hemolysis and shown in Figure 2. H_2_O_2_ significantly induced oxidative stress in erythrocytes with dose and time-dependent manner. Over 4 h, there was no hemolysis in erythrocytes without H_2_O_2_ treatment. Erythrocytes treated with 100 and 200 mM H_2_O_2_ showed significant hemolysis after 3 h treatment (*p* < 0.01). However, erythrocytes treated with 300, 400, and 500 mM H_2_O_2_ displayed significant hemolysis after 1 h exposure (*p* < 0.01). Since there was significant increase in hemolysis rate after 1.5 h treatment of erythrocytes with 400 and 500 mM H_2_O_2_ (*p* < 0.01) and no significant difference of hemolysis rate was observed between 400 and 500 mM H_2_O_2_ treatment for 1.5 h, the 400 mM concentration and 1.5 h exposure were selected for further investigation of biological effects of orientin and luteolin.

### 3.2. Effects of Orientin and Luteolin on Hemolysis of Erythrocytes

**Figure 3:**
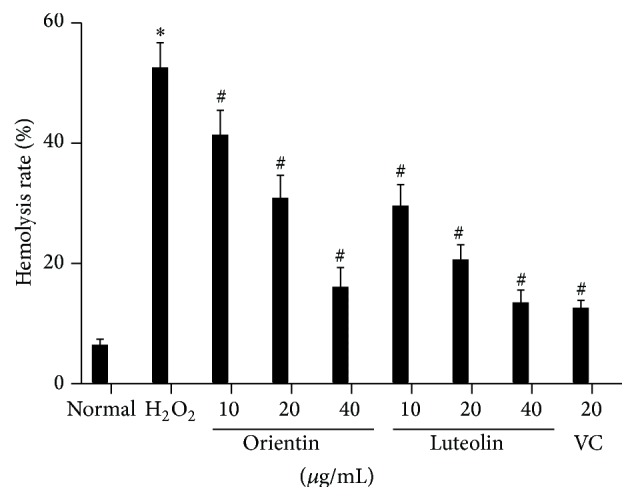
Both orientin and luteolin attenuated hemolysis rate of erythrocytes. Oxidative stress induced significant increase in hemolysis rate of erythrocyte. However, both orientin and luteolin ameliorated hemolysis in a dose-dependent manner. Data are presented as mean ± SD from 10 individual experiments. *∗* represents *p* < 0.05 compared to normal control and # indicates *p* < 0.05 compared to oxidative stress erythrocytes.

The effects of orientin and luteolin on hemolysis rate of erythrocytes were shown in Figure 3. Oxidative stress induced significant hemolysis of erythrocytes compared to normal erythrocytes (*p* < 0.01). Both orientin and luteolin significantly attenuated hemolysis of erythrocytes in oxidative stress group in a dose-dependent manner and at the concentrations of orientin (10 or 20 *μ*g/mL) were higher than the same concentrations of luteolin (*p* < 0.05). However even with highest concentration of orientin and luteolin (40 *μ*g/mL), hemolysis rates were still higher than normal erythrocytes but at the same level of vitamin C treated erythrocytes.

### 3.3. Effects of Orientin and Luteolin on Antioxidant Enzymes, ATPase, ROS, and MDA of Human Erythrocyte Exposed to H_2_O_2_

**Figure 4:**
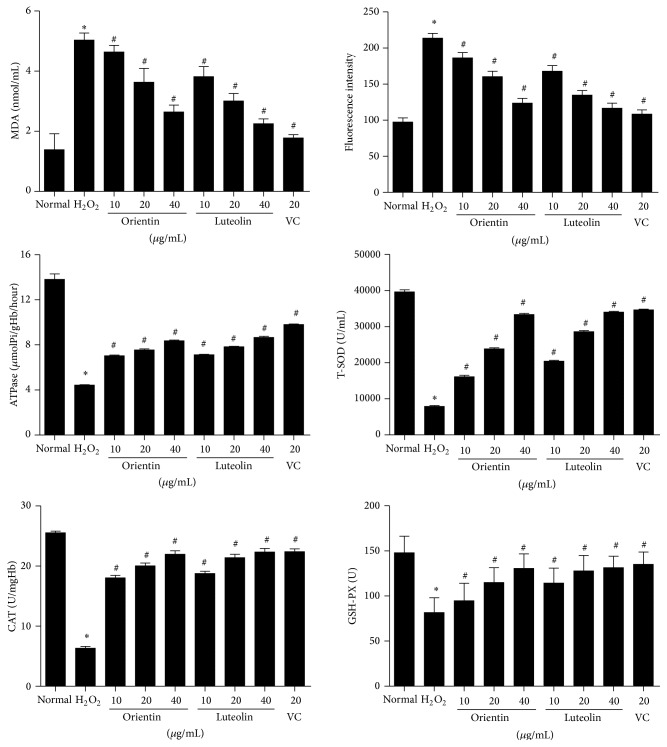
Regulations of MDA, ROS, ATPase, total SOD, CAT, and GSH-PX in human erythrocytes under oxidative stress by orientin and luteolin. The panels represent the results of MDA, ROS, ATPase, total SOD, CAT, and GSH-PX with treatment of orientin and luteolin under oxidative stress, respectively. Data are presented as mean ± SD from 10 individual experiments. *∗* represents *p* < 0.05 compared to normal control and # indicates *p* < 0.05 compared to oxidative stress erythrocytes.

When erythrocytes were treated with H_2_O_2_, there were significant increases in ROS and MDA content (Figure 3). However, when erythrocytes were incubated with either orientin or luteolin with H_2_O_2_, there were gradual decreases in both ROS and MDA content in a dose-dependent manner and at the concentrations of orientin (10 or 20 *μ*g/mL) that were higher than the same concentrations of luteolin (*p* < 0.05). Even with highest concentration of either orientin or luteolin, they could not reduce ROS and MDA content to normal erythrocyte levels. Moreover, H_2_O_2_ protected antioxidative enzymes (SOD, CAT, and GSH) and ATPase in erythrocytes and both orientin and luteolin could recover these enzymes activities to almost the levels of VC treated erythrocytes and at the concentrations of orientin (10 or 20 *μ*g/mL) were lower than the same concentrations of luteolin (*p* < 0.05) (Figure 4).

### 3.4. Effects of Orientin and Luteolin on Surface Morphology and Skeleton Structure of Erythrocytes

**Figure 5:**
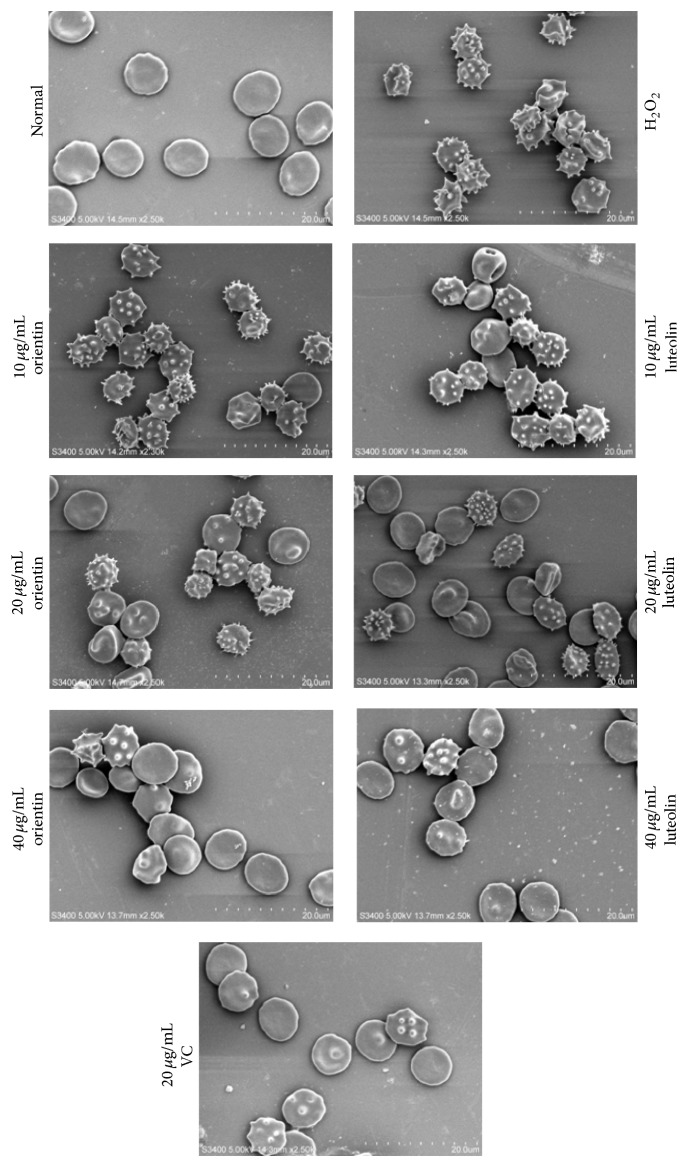
Effect of oxidative stress with treatment of orientin and luteolin on erythrocyte cell surface structure. Typical erythrocyte cell surface morphologies were observed under SEM with magnification of 2500x. The treatments were indicated in the figure. The bar in the picture represents 20 *μ*m.

**Figure 6:**
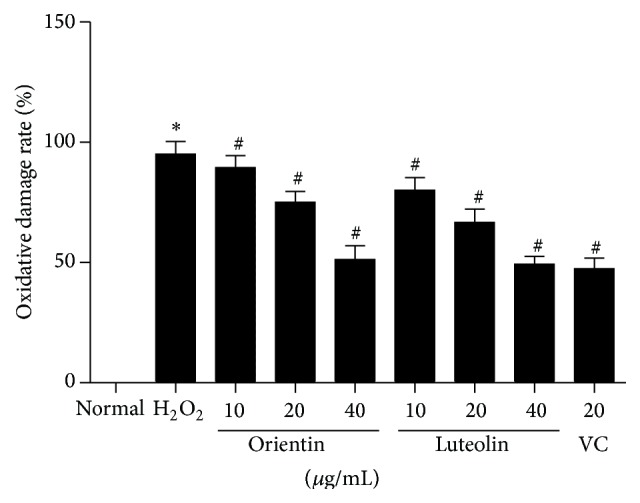
Both orientin and luteolin attenuated oxidative damage rate of erythrocytes. Oxidative stress induced significant increase in oxidative damage rate of erythrocyte. However, both orientin and luteolin ameliorated oxidative damage in a dose-dependent manner. Data are presented as mean ± SD from 10 individual experiments. *∗* represents *p* < 0.05 compared to normal control and # indicates *p* < 0.05 compared to oxidative stress erythrocytes.

**Figure 7:**
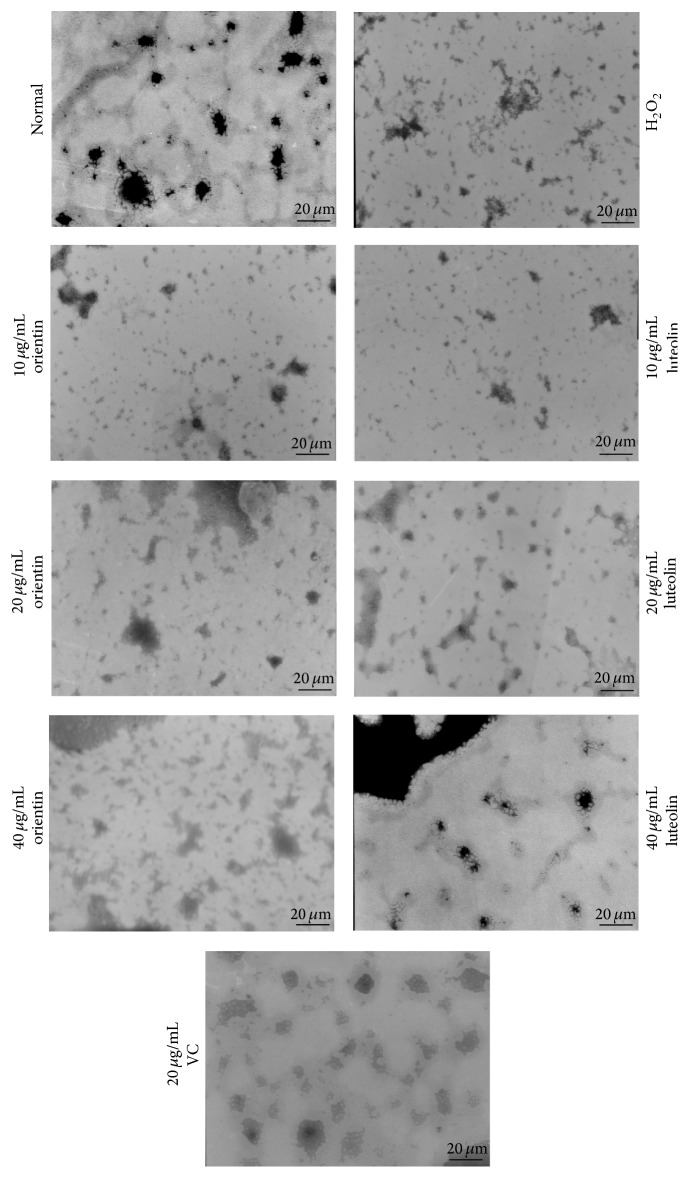
Effect of oxidative stress with treatment of orientin and luteolin on erythrocyte cellular structure. Typical erythrocyte cellular structures were observed under TEM with magnification of 30000x. The treatments were indicated in the figure. The bar in the picture represents 20 *μ*m.

Normal erythrocyte surface was smooth and there were no spike-like processes extending out from surface. However when erythrocytes were exposed to H_2_O_2_ erythrocytes, significant amounts of spike-like processes were extended out from the surface. When these erythrocytes were treated with 20 *μ*g/mL of either orientin or luteolin, the amounts of spike-like processes were significantly reduced (Figures 5 and 6). Vitamin C served as positive control and had the same effect on erythrocyte surface morphology as orientin or luteolin. In addition, when erythrocytes were treated with H_2_O_2_, no network-like structure appeared. The J point was not clear and the spectrin tetramer (SP4) assembly was fractured. After treatment with 20 *μ*g/mL of either orientin or luteolin, the erythrocyte membrane skeletal J point was gradually restored, and the SP4 assembly gradually became complete (Figure 7). The same concentration of vitamin C treatment had the same effect on erythrocyte skeletal structure.

## 4. Discussion

Human erythrocytes only have certain lifespan in the blood because they do not have nuclei and mitochondria. Moreover, erythrocytes were exposed to large amount of free radicals that are circulated in the blood. Therefore, oxidative stress of erythrocytes has been proposed as one of the aging mechanism of human erythrocytes [18]. H_2_O_2_ is a free radical and has been widely used to induce oxidative stress* in vitro* cell experiment. H_2_O_2_ can easily pass through the cell membrane and form highly reactive free radicals with iron molecule by the Fenton reaction [19], which leads to structural and functional damage of the normal cells. This study showed that erythrocyte hemolysis rate of 400 mM H_2_O_2_ at the duration of 1.5 h was 52.51 ± 4.22%, reaching the half damage and having no significant difference with 500 mM H_2_O_2_, which was thus selected for further research. Since erythrocytes lack the nuclei and mitochondria, erythrocytes have cellular membrane deformation, suspension instability, and osmotic fragility. Therefore, its function and structural integrity are sensitive to changes of endogenous and exogenous reactive oxygen content. Our results are consistent with the proposal that oxidative stress causes erythrocyte hemolysis.

From oxidative stress hypothesis, any chemicals that can reduce oxidative stress would have ability to reduce erythrocyte injury and prolong its lifespan in the blood. Shortage of erythrocyte lifespan has been demonstrated to associate with anemia, which is a life threaten condition [20]. Several natural products from the traditional Chinese medicine or native medicine are used commonly to treat anemia with excellent outcomes. For example, one study demonstrated the protective effects of quercetin against H_2_O_2_-induced oxidative damage of erythrocytes. Although they showed similar antioxidative activity of quercetin, the erythrocyte hemolysis rate and MDA content are not consistent with the findings in the current study [13]. These differences could be due to the concentrations and duration of used H_2_O_2_. Moreover, the extract of* Panax japonicus* was also able to attenuate hemolysis of erythrocytes through inhibition of lipid peroxidation of erythrocytes [21, 22]. Furthermore, vitexin was documented to prevent erythrocytes from oxidative damage induced by ROS, preserve antioxidant enzyme activity, and maintain the integrity of erythrocyte morphology and membrane skeletal ultrastructure [23]. Therefore, other flavonoids such as orientin and luteolin may also have similar antioxidative activity.

Orientin belongs to the family of flavonoid glycosides [9] and has abilities to reduce oxidative stress and apoptosis as well as decrease fat and blood sugar [24, 25]. In ischemic-reperfusion model of heart, orientin was able to protect ischemic injury of cardiomyocytes [12] and inhibited apoptosis of these cells [10]. This is consistent with our findings that orientin had antiapoptosis activity in ischemia-reperfusion injury by neutralizing superoxide anions and hydroxyl radicals* in vitro* [26]. Luteolin belongs to the flavonoid aglycone class [27] and has various activities such as antioxidative, anti-inflammatory, and anticancer activities [28–30]. Especially for its antioxidative activity, luteolin glycosides could prevent myocardial cell injury from H_2_O_2_-induced oxidative stress through increasing SOD activity and decreasing the amount of MDA and ROS [11].

Although orientin and luteolin have similar structure of a 15-carbon skeleton, which consists of two phenyl rings (A and B) and a heterocyclic ring exception of a C-glycosides linked to glucose of the A ring, our findings indicate that luteolin have better antioxidative activities than orientin in 10 and 20 *μ*g/mL dose groups, which could be related to structural differences between these two compounds. However whether these differences are related to the C-glycoside linked to glucose of the A ring remains to be investigated.

## 5. Conclusion

Both orientin and luteolin could attenuate oxidative stress of erythrocytes through the decrease of the level of ROS and the radicals generation, thereby protecting the antioxidative enzymes and then reducing the damage to structure of cell membrane in H_2_O_2_ induced oxidative stress model of erythrocytes.
